# Formative Evaluation to Build an Online Parenting Skills and Youth Drug Prevention Program: Mixed Methods Study 

**DOI:** 10.2196/14906

**Published:** 2019-11-05

**Authors:** Lawrence Matthew Scheier, Karol L Kumpfer, Jaynie Litster Brown, QingQing Hu

**Affiliations:** 1 LARS Research Institute Scottsdale, AZ United States; 2 University of Utah Salt Lake City, UT United States; 3 Strengthening Families Program, LLC Salt Lake City, UT United States; 4 Division of Public Health, University of Utah Salt Lake City, UT United States

**Keywords:** formative evaluation, parenting skills, drug prevention, focus groups, key stakeholders, consumer preference survey, internet intervention

## Abstract

**Background:**

Family-based drug prevention programs that use group-based formats with trained facilitators, such as the Strengthening Families Program (SFP), are effective in preventing underage drinking and youth drug use. However, these programs are resource-intensive and have high costs and logistical demands. Tailoring them for Web-based delivery is more cost-effective and makes it easier to scale these programs for widespread dissemination. This requires the active involvement of all key stakeholders to determine content and delivery format.

**Objective:**

The aim was to obtain consumer, agency stakeholder, and expert input into the design of a Web-based parenting skills training and youth drug prevention program.

**Methods:**

We conducted 10 focus groups with 85 adults (range 4-10, average 8 per group), 20 stakeholder interviews with family services agency staff, and discussed critical design considerations with 10 prevention scientists and e-learning experts to determine the optimal program content and technology features for SFP Online. Focus group participants also answered survey questions on perceived barriers to use, desired navigational features, preferred course format, desired content, preferred reward structures, course length, interactive components, computer efficacy, and technology use. Descriptive statistics were used to examine consumer characteristics; linear regression was used to examine relations between SFP exposure and four continuous outcome measures, including desired program content, interactive technology, and concerns that may inhibit future use of SFP Online. Logistic regression was used as a binary measure of whether consumers desired fun games in the SFP Online program.

**Results:**

Three broad thematic categories emerged from the qualitative interviews enumerating the importance of (1) lesson content, (2) logistics for program delivery, and (3) multimedia interactivity. Among the many significant relations, parents who viewed more SFP lessons reported more reasons to use an online program (beta=1.48, *P*=.03) and also wanted more interactivity (6 lessons: beta=3.72, *P*=.01; >6 lessons: beta=2.39, *P*=.01), parents with less interest in a mixed delivery format (class and online) reported fewer reasons to use the online program (beta=−3.93, *P*=.01), comfort using computers was negatively associated with concerns about the program (beta=−1.83, *P*=.01), having mobile phone access was related to fewer concerns about online programs (beta=−1.63, *P*=.02), willingness to view an online program using a mobile phone was positively associated with wanting more online components (beta=1.95, *P*=.02), and parents who wanted fun games wanted more interactivity (beta=2.28, *P*=.01).

**Conclusions:**

Formative evaluation based on user-centered approaches can provide rich information that fuels development of an online program. The user-centered strategies in this study lay the foundation for improving SFP Online and provide a means to accommodate user interests and ensure the product serves as an effective prevention tool that is attractive to consumers, engaging, and can overcome some of the barriers to recruitment and retention that have previously affected program outcomes in family-based prevention.

## Introduction

### Background

Youth alcohol and drug use continue to be top public health priorities. This emphasis stems from recognition that experimental drug use that often coincides with adolescent rebelliousness can easily transform into addiction if left unabated. Numerous trials have now shown that family-based drug prevention programs are an effective first line of defense preventing underage drinking and youth drug use [1-3]. The evidence base also reinforces the utility of combining parenting skills training with program content that emphasizes youth drug prevention [4]. An important consideration in this approach is that family dynamics, and especially parent-child social interactions, play a crucial role in a child’s developmental outcome [2,5-7]. Most—if not all—of these programs have their conceptual roots in ecological [8], social interactional [9], and transactional [10] models of human development. All these approaches underscore the close interpersonal alliance formed between parent and child and its incipient role in developmental outcomes. Regardless of conceptual underpinnings, most family-based programs strongly reinforce that parents shape their child’s behavior from a very early age.

The Strengthening Families Program (SFP) is one example of a parenting skills training program that combines youth drug prevention in a group-based, facilitator-led format for children aged 3 to 17 years. The program was first tested with 14 sessions designed to improve parenting skills among drug-addicted adults in treatment settings [11,12]. Since then, the program has been retooled for universal settings [13], applied to young children [14], delivered in rural settings [15], tested in urban schools [16], culturally adapted [17,18], and examined using rigorous randomized controlled trials in international settings [19,20]. There is now also evidence the program can promote parent-child reunification in child welfare settings [21] and produce other societal and economic benefits [22].

In keeping with well-established research traditions, the program’s core components draw from family systems theory [23], relationship enhancement approaches [24], family-based therapeutic approaches [25], behavioral parent training [9], and social learning theory [26]. Collectively, these theoretical linchpins support the goal of teaching parents how to bond with their child, use more effective ways to communicate with their child, set reasonable boundaries and limits (controls and restrictions), reward their child for good behavior, and monitor their child’s activities [27]. The child component addresses social and personal skills that will help them refuse negative peer pressure to use alcohol and drugs and improve their personal self-management skills. Children also practice ways to increase social-emotional regulation and impulse control and acquire better problem-solving and effective communication skills.

Similar to many other family-based programs, parents and children receive separate instruction in the first hour and then join together to practice and receive feedback on newly learned skills during a second hour. This portion includes supportive role play and games that the parent and child play together, which encourages parents to implement nonjudgmental dialog and increase parent-child attachment. Behavioral reinforcement techniques, such as situational role play and behavioral rehearsal with positive feedback given to families by highly trained facilitators, are a hallmark feature of SFP. The program uses curriculum guides, homework, and workbooks that can be practiced in the home to reinforce lesson material. Providing family meals before each weekly session, offering childcare for young siblings, and assisting with transportation are implemented to address attendance barriers that potentially inhibit participation and retention over time.

### Internet-Delivered Parenting Programs

Notwithstanding evidence of their overall effectiveness, family-based programs remain resource-intensive with high costs and logistical demands. Any attempt to scale them for widespread dissemination has to address these implementation demands. In recent years, internet-based behavioral interventions have been making tremendous strides as alternatives to programs that customarily use face-to-face delivery, presenting a promising avenue for delivery [28,29]. Web-based programs ensure standardized delivery and implementation fidelity, are convenient for end users, and are self-paced, cost-efficient, and reduce social or personal demands. Some parenting skills programs have taken on this challenge, and a few internet-based programs have produced promising findings underscoring their efficacy [30-35]. Two programs that have successfully migrated to the Web include Familias Unidas [30,31] and Triple P Online [32,33]. Familias Unidas targets parents with goals of preventing substance use, sexual risk behaviors, and sexually transmitted infections in high-risk youth from immigrant Hispanic and Latino families; Triple P is a population-based intervention that targets families with young children with early-stage conduct disorder or behavioral or emotional problems. Both programs use formative evaluation strategies in the process of developing an eHealth prototype. These efforts included focus groups to identify the program features desired by consumers and discussed with families novel ways to structure program content when delivered on a Web platform.

### Challenges With Web-Based Delivery

Repackaging group-based, facilitator-led programs for Web delivery faces several formidable challenges. To begin with, a program developer must consider how to preserve the active ingredients of a program as it is transitioned to a Web-based platform. This entails finding ways to transform behavioral practice and positive reinforcement techniques into digital content that preserves the integrity of the intervention strategies. Then developers must consider finding the proper dosage and session length, particularly because internet-based programs are traditionally shorter than in-person, group-based formats. Developers must also find ways to assess exposure, including engagement, and use this information to establish a metric for fidelity of implementation and adherence—two important components related to program efficacy [36-38].

Engagement can be defined as the adoption of the program by consumers, and then continued use can be measured through session exposure. In both cases, recruitment to participate and retention throughout delivery in family-based programs have faced several hurdles [39-41]. Indeed, reviews of recruitment and retention in family-based programs highlight the problem of attrition, with a host of reasons cited by participants for their noncompliance [41,42]. Reasons for failing to attend or complete the program include that parents may not find the program accessible or they perceive the intervention demands as burdensome (ie, lengthy sessions) or intimidating. In the traditional group setting, additional logistical barriers to participation include transportation to and from the facility, costs associated with childcare if not provided by the program, and time away from family responsibilities [43]. Scheduling demands can also interfere with participation. In some cases, parents have expressed reluctance to attend because of stigma, loss of privacy, or the appropriateness of the intervention for their essential needs [30,41]. Regardless of their stated reasons, dropping out of the intervention lowers dosage and adversely affects program outcomes, reduces power, and limits external validity.

### Involving Users in Program Design

Transparency in the process of meeting these challenges would provide a template to guide future research examining the design and implementation of Web-based parenting skills training and youth drug prevention programs [44]*.* Participatory action research strategies in the form of consumer preference studies represent one way to address the concerns that arise from the deployment of Web-based programs [45,46]. Consumer preference studies are a mainstay of community-based research, as they seek greater input from consumers in all phases of a program from its early conceptual stages through development, implementation, and even dissemination. There is now growing evidence that consumer preferences can be quite fruitful when designing programs for youth, especially those focusing on mental health [47]. The strategy of gleaning information from consumers was also the tactic used by the developers of Familias Unidas [30] and Triple P [32-33] when they solicited consumer’s input before the developers designed a Web-based delivery system for their family programs. By allowing participants to contribute and provide input in the early program design stages as part of “collective making,” consumers feel valued as co-creators [48]. The goal here is to have the target consumer population take a more vested interest in program development, feel empowered, and take ownership, leading the developer to construct a product that is more reflective of the target consumer’s preferences and needs [49]. Motivation to attend sessions should be higher among consumers when they feel the session content and delivery format are well designed and congruous with their needs.

This study uses a mixed methods formative evaluation design to explore consumer preferences in the prototype build stage for SFP Online*.* In choosing to develop an online version, several pressing questions needed to be addressed, including whether consumers felt the group-based format could be transformed in a way to maintain the active ingredients with no loss of fidelity in a digital environment. We also wanted to know what levels of interactivity and what type of program content are needed to offset factors that contribute to noncompliance. We applied both formative and summative strategies to obtain this information before developing and testing a prototype. We also posed specific questions to consumers regarding the feasibility, perceived utility, technology acceptance, desired instructional format (ie, screen layout and navigational features), and other utilization factors that could influence engagement. This type of in-depth feedback is essential during the development process because it can be a foundation for the design of intervention modules and help structure their delivery. Although we solicited and received input from both parents and youth, we only focus on the adult consumer responses in this study.

We also extended prior work by including agency staff (stakeholders) and subject matter experts because they often have tangible insight that can shed light on and give shape to the product’s functionality. Agency staff who routinely work with families offering services may have their finger on the pulse of what families want and need and may also recognize some of the barriers to attendance that affect program outcomes. Experts can weigh in on how to maintain close alignment with the developmental and family-based theories that drive most parenting skills training interventions. As detailed subsequently, a second angle that e-learning experts can weigh in on includes integrating various instructional design principles into a serious educational game to increase the program’s overall attractiveness. In addition to the formative strategies, we used a consumer preference survey administered to focus group participants, which is explained in detail.

## Methods

Table 1 shows the study design and gives a brief description of each arm corresponding to the formative and summative evaluation research plan. This study was part of a Phase I NIH-funded Small Business Innovative Research grant (NIDA; R43DA046238-01) and the study was approved by the Heartland IRB located in Belleville, IL (HIRB Project #180213-187) prior to engaging in any scientific work.

**Table 1:** Research plan for the different study arms.

Study arm	Sample size	Focus	Comments
Focus groups	85 adults in 11 separate groups	Necessary steps for translation of group-based program to a Web platform	Questions probed active ingredients, interactivity, cellular phone and computer efficacy, proposed content, games, and ways to connect parent and youth lessons
Consumer preference survey	85	Barriers and facilitators, computer technology familiarity, desired content and materials, interactivity, and desire for social media connections	45 questions assessing past experience with the Strengthening Families Program assessing whether prior program exposure guides consumer preferences for an interactive, multimedia Web platform
Key stakeholder interviews	20 agency staff	Suitability of Web platform for clientele, assessing required changes in content and delivery methods, whether program would be engaging (interactivity) and fit the agency goals	Semistructured interview with questions provided in advance, then teleconference call to expand on answers
Expert interviews	10 (5 prevention scientists, 5 e-learning experts)	Two themes: prevention science to bolster core active ingredients of program and e-learning emphasis to include recent technology and its influence on learning and behavior change	Semistructured interview with questions provided in advance, and then phone or video interview used to expand on answers

### Focus Groups

We recruited 85 adults from nine geographically dispersed agencies in the US covering the western portion (NM, CA, WA, UT, and NV), the middle portion (KS, IN, and OH), the eastern seaboard (NC and NY), and the south (TX). These agencies were representative of the target agencies where SFP Online could be distributed, and they were willing to participate in the formative evaluation. The agencies conducted the focus groups between May and early June of 2018. Each agency had participated in prior SFP implementation training using the traditional group format or had implemented the DVD curriculum used in conjunction with group classes. An email was sent to all the agencies outlining the scope of the project and the requirements for participation. After the initial contact, we sent a memorandum of agreement to each agency outlining the participant recruitment requirements (emails, phone calls, and posted flyers) and what family participation would entail, including a description of incentives that would be provided to both the individuals and the agency.

We gauged the number of groups (approximately 4 to 10 members in each group) based on recent studies that confirmed thematic sufficiency with nonprobability sampling strategies can be achieved with three to six focus groups [50] and also accounted for potential dropout after recruitment. The inclusion criteria stipulated that parents (including guardians and caregivers) and youth had attended an SFP class or watched the SFP DVD. This requirement was intended to solicit input from families who had sufficient knowledge of the programs’ core content and could comment on the suitability of an online version, make suggestions for future content, and discuss the utility and feasibility of a Web-based version.

Each focus group lasted between 60 and 90 minutes and involved a moderator; refreshments were provided at the midpoint. Agency staff assisted in obtaining written informed consent, conducting the groups, and collecting survey materials. Moderators were given a seven-page instructional guide outlining how to conduct the group, preserve confidentiality, and solicit balanced input from the group members. Moderator probes queried the parents’ computer technology and mobile phone usage, their video game experience, desired program content, instructional materials, navigational features, interactive components, and whether parents wanted asynchronous social media connections (ie, blogs, chat, discussion board). Members of the research team were present during one of the focus groups and listened to others via GoToMeeting Web conferencing.

### Key Informant Interviews

We also conducted 20 key informant semistructured interviews by teleconference with agency staff primarily drawn from the same agencies that participated in the focus group. One exception was a new agency in MA that participated in the interviews. One agency staff from each site participated, except for NY, UT, and KS, where more than one staff member was interviewed. Agency staff had been previously trained to deliver SFP as part of activities distinct from this project; they also provide, as part of their regular duties, additional psychoeducational and support services to the families. Their input was intended to address whether they felt the intended Web-based program could retain the core instructional strategies, how to structure the instructional modalities so they would be engaging, and what technology design features would be appropriate for the target families. Before the phone call, each stakeholder was provided a 20-item questionnaire with specific probes intended to elucidate their involvement with SFP, their organizational role, the demographic profile of the agency clientele (racial composition, income, risk level), their perceived feasibility and utility of implementing SFP Online, and the essential program components and skills training they felt should be included.

### Expert Panel

We recruited 10 subject matter experts to weigh in on prototype development. Five were prevention scientists who we asked to address the procedures for translating core active ingredients into online programming and ways to ensure engagement. An additional five e-learning experts shared ideas on different ways to capitalize on current trends in multimedia interactive programming and whether this should influence the design of SFP Online. A brief survey was emailed to the experts before the teleconference interview, and their written responses helped guide the interview. The first part included 10 brief questions intended to elucidate their experience designing interactive programs. This was followed by 19 interview probes addressing the feasibility of SFP Online, implementation strategies, use of live family coaches, social media, interactive activities to promote family problem-solving skills, dashboard and navigation features that might be attractive, methods to stimulate engagement and increase session exposure, and perceived barriers or problems when implementing eHealth programs (eg, recruitment, retention, session length, interactivity, and structuring programmatic content).

### Consumer Preference Survey

Parents in the focus groups also filled out a 45-item consumer preference survey. Following completion, the anonymous surveys were collected by agency staff; placed in sealed, preaddressed envelopes; and mailed to the research team. Table 2 shows the survey items (predictors) that were used in the summative analyses to examine consumer preferences. Additional outcome measures are explained subsequently. Fixed-choice or dichotomous yes or no response formats were used for some questions; the balance used five-point Likert response formats.

**Table 2:** Predictor measures from the consumer preference survey.

Survey question	Response format^a^
How many Strengthening Families Program (SFP) DVD lessons did you watch?	0, 1-3, 4-6, 7-9, 10-11
How comfortable are you using a computer?	Very, somewhat, not very
Would you prefer doing the SFP course online, or would you rather attend a class?	Online, attend class, undecided
Would you prefer to track your skills practice on your computer instead of a paper handout?	Yes, maybe, probably not, no
Are you interested in using a game-like online version of SFP?	Yes, maybe, not really, not interested at all
Would you practice the skills at home without a live family coach to remind you?	Yes, no, maybe, maybe with points
Do you have access to a computer that connects to the internet?	Yes/no
Would you use SFP Online even if you also took a class?	Yes, maybe, probably not, not
Do you have a mobile or smartphone where you could view SFP Online?	Yes/no
If you have a smartphone, would you view SFP Online on your phone?	Yes/no
If you could access SFP Online, how likely are you to record your home practice assignments?	Likely, somewhat likely, somewhat unlikely, unlikely
How many lessons should be included in SFP Online?	1-5, 6-10, 11-15
How long should each individual lesson be?	6-8, 9-12, 13-20, 21-30, >30 minutes
Would including fun games in SFP Online help your family learn new skills?	Yes, maybe, probably not, no
Would you like family members to earn reward points after completing SFP assignments?	Yes, maybe, probably not, no
Which of the following would you prefer in a family game?	Points to reward progress, experience points to move up a level in game, both
Would you like SFP Online games to include a family competition for points when you practice the skills?	Yes/no
Do you think you would practice the skills at home without a live family coach to remind you?	Yes, no, maybe, maybe, if we earned points for doing it

^a^Some variables recoded to avoid sparse cells. Age, gender, and race/ethnicity also asked. Race/ethnicity categories included African American or black, Asian, American Indian, Pacific Islander or Native Hawaiian, Alaska Native, white, Hispanic or Latino, and more than one race.

### Qualitative Coding Schemes

Digital audiotapes from the focus groups and interviews were professionally transcribed verbatim, and the content of this material was then examined using thematic analysis as a research tool. This approach breaks down the open-ended answers into smaller units so the researcher can actively identify consistent and meaningful patterns in the text [51]. We used a data-driven inductive approach similar to grounded theory [52,53] to quantify themes based on explicit content analysis. For example, thematic content from the focus groups might reference the word “games” used in the context of programmatic features that will attract youth and contribute to engagement. Likewise, stating the need for interactive components modeled after a coach would be considered a theme (ie, “the program needs a coach”). Frequency of how often a word or phrase appeared in different contexts provided a quantified metric of thematic sufficiency. We repeated this coding procedure across all groups with two members of the investigative team descriptively tabulating key terms and phrases verbatim and then synthesizing across groups until thematic saturation was obtained. The coders read the transcripts and compiled their thematic results independently, and then convened for discussion of emerging themes. The results would then be summarized and tabulated into a product specification plan, a written document presented to the product design team before the alpha prototype build.

### Consumer Preference Survey: Outcome Measures

The survey included additional items assessing issues that might arise in using SFP Online, which were modeled as outcomes. Seven items assessed technology barriers that might present concerns when using SFP Online, including data plans, visual content, screen size, and navigation, with responses coded on a five-point Likert scale ranging from not at all concerned to extremely concerned. An additional 20 items assessed what subject material parents felt was important to include in the online version (eg, brain development, mindfulness, communication skills, giving compliments, and monitoring) with responses coded on a five-point Likert scale ranging from very important to not at all important. An additional 16 items assessed the importance of different program components for learning parenting skills (eg, reporting progress, short videos, family goals, virtual coach, and tracking progress) with responses coded on a five-point Likert scale ranging from very important to not at all important. A 10-item scale assessed reasons for using SFP Online (eg, self-paced, review lesson materials, monitoring family progress, and tracking family performance) with responses coded on a five-point Likert scale ranging from very important to not at all important. A single item asked parents if they were interested in a game-like version of SFP, with responses recoded as yes or no.

Both factor analysis and estimates of internal consistency indicated low reliability and lack of conceptual purity in the outcome scales. This is likely because parents treated each subset of items as a checklist, and this prevented us from obtaining homogenous unidimensional scales. As a result, we created unit-weighted indexes to represent the different technological, logistic, programmatic, and motivational reasons for using SFP Online. We dichotomously recoded the Likert response scales to 1 or 0 by collapsing “not at all concerned” and “slightly concerned” to 1 and coded the remaining response categories to 0. Likewise, we collapsed “very important” and “important” to 1 and all other responses to 0. For all analyses, the significance level was set at *P*<.05 with two-sided tests.

## Results

### Adult Focus Group Characteristics

Eighty-five parents attended the 10 focus groups (77% female, 65/85). Their age breakdown included 11 parents (13%) between 18 and 31 years, 43 parents (51%) between 32 and 45 years, 19 parents (22%) between 46 and 52 years, 10 parents (12%) between 53 and 66 years, and 2 grandparents (2%) 67 years and older. In all, 56 (66%) were white, 8 (9%) were Native American or American Indian, 5 (6%) were Hispanic or Latino, 4 (5%) were African American, 9 (9%) were mixed race, 2 (2%) were Asian, and 1 (1%) indicated “other.” The family service agencies served a fairly homogeneous clientele, who were relatively poor, socially marginalized, experiencing family distress, and characteristically low in education. Therefore, we did not ask about their socioeconomic status using income or education.

Of the 85 parents, 79 (93%) said they had computer access with internet connection, and 69 (82%) said they were comfortable using computers, 11 (13%) said they were somewhat comfortable, and 5 (6%) said they were not comfortable. As anticipated, all the parents had some exposure to the SFP traditional group-based classes. Twenty-nine (34%) took a class with their family. Twenty (24%) viewed the SFP DVD video clips in a class setting, 21 (25%) viewed the DVD at home, and 11 (13%) viewed the SFP DVD with a coach. Thirty-three (39%) parents attended between zero and three lessons, 11 (13%) attended between four and six lessons, and 40 (48%) attended more than six lessons. Twenty-two (26%) parents said they preferred an online class, 35 (41%) said they wanted to attend an in-person class, and the remaining 23 (27%) were undecided between the two. Seventy-four (87%) parents said they would view SFP Online if it was available on a smartphone. When asked about including games in the new online version, 4 parents (4%) said they were not at all interested in games, 10 (12%) said they were slightly interested, 41 (48%) said they might be interested, and 30 (35%) said they would be very interested.

### Qualitative Analyses

A total of 241 keywords were culled from the thematic content analysis of the focus groups and interviews. Two coders conducted the content analysis and reduced the pool of open-ended answers to 86 distinct keywords. An example of a word that parents provided that was highly prevalent was “activities,” and the corresponding commentary included “parent-and-child activities to do together.” Another example was “challenges,” and the commentary included “ability to customize challenges” and “challenges that spark our interest.” We then tallied the keywords by frequency of their use with the most popular terms, which included “games” (n=55), “unique” (n=40), “fun” (n=15), “challenges” (n=14), “points” (n=11), and “rewards” (n=9). The remaining terms had frequencies in the single digits. Using an inductive procedure, we then summarized these terms into three major categories: program content, logistics, and interactivity/engagement. Program content encompassed teaching points and lesson plans (eg, discussing risk and protective factors for drug use, practicing family management skills, and teaching families how to create strong bonds), and referenced specific SFP core activities (eg, communication boulders, mindfulness, and automatic negative thoughts).

Logistics encompassed program delivery features, such as what will make SFP Online engaging (technology and navigation control) and encourage future continued use (eg, natural-sounding voices, customization and feedback, accessibility on multiple devices, animation, and realistic role play). Interactivity and engagement encompassed multimedia features of the program (eg, avatars, chat or threaded discussion boards, expert coaching, pop-up notifications, and weekly expert blogs). Textboxes 1 to 3 provide several examples corresponding to each category created from the three different formative evaluation strategies.

**Thematic content analysis: SFP (Strengthening Families Program) Online summary categories for focus groups.**

Content

1. Explain importance of sincere compliment (brain triggers); show Emotional Bank Account
2. Explain five steps of a reinforcing compliment
3. Keep a 4:1 ratio of compliments to corrections
4. Click to create compliments you can give family members
5. Short, realistic video clips
6. Download using SFP handouts

Logistics

1. Clear, simple navigation tools; effective search bar
2. Bright colors; animation; lots of graphics; no clutter
3. Customizable dashboard
4. App for tracking compliments
5. Interface linking parent and child responses
6. Points for completed lessons

Interactivity/Engagement

1. Virtual, customizable family coach who can ask questions, give advice, invite family to practice
2. Customizable Cheering Squad
3. Rewards for learning and practicing
4. Allow optional competition
5. Send chat messages to a family member (parent discussion board)
6. Use Scoreboard to track and see if achieving personal SFP goals
7. Earn “practice coins” to “buy” accessories for avatar and coach

**Thematic content analysis: SFP (Strengthening Families Program) Online summary categories for key informant interviews.**

Content

1. Allow families to choose lessons; explain prerequisites
2. Ability to download and use SFP handouts
3. “Yes, but...” page to resolve concerns with a virtual family coach
4. Page with additional info: “To learn more, click here...”

Logistics

1. Simple, short, text language
2. Audio for nonreaders
3. Interface between parents and children to send messages
4. Frequently asked parenting questions with answers

Interactivity/Engagement

1. Text message reminders
2. Reward family completion of a lesson visually on dashboard (eg, assemble a family photo puzzle into a frame by joint practicing of skills)
3. Enable parent discussion board (eg, assemble a family photo puzzle into a frame by joint practicing of skills)

**Thematic content analysis: SFP (Strengthening Families Program) Online summary categories for expert interviews.**

Content

1. Teach core essential SFP Skills that target key risk and protective factors
2. Include on-going review
3. Earn points for skill practice
4. Reward extra points and give congratulations on completion
5. Provide family and individuals Certificate of Completion

Logistics

1. Ability to practice and get quick feedback.
2. Low-stakes failure with immediate correcting feature
3. On sign up, receive a Family Page plus subpage for each child
4. Site autoreports results completed and skills practiced
5. Mobile phone accessible

Interactivity/Engagement

1. Customizable Family page with name, banner, motto, Dashboard
2. Customize learning modules to fit the age of children
3. Allow parents and youth to choose lesson topics; explain needed lesson prerequisites for each skill
4. Reward family goal setting

### Consumer Preference Survey: Gender, Race, and Age Differences

There were no gender differences in the categorical measures assessing parents’ SFP course exposure, technology readiness, or course content preferences. Younger parents were more likely to want to view SPF Online using their mobile phone (χ^2^_1_=4.0, *P*=.04, φ=−0.22), were more likely to want to track their skills practice on a computer (χ^2^_2_=8.4, *P*=.01, φ=0.31), and were more likely to want a coach (χ^2^_2_=7.6, *P*=.02, φ=0.29). White parents (compared with all other race/ethnic groups) viewed more SFP lessons (χ^2^_2_=7.1, *P*=.03, φ=0.29) and preferred doing the classes online (χ^2^_2_=6.7, *P*=.03, φ=0.28). There were no significant race/ethnicity, age, or gender differences for desiring a gamified version of SFP Online. There were also no significant demographic differences in the mean scores for the four outcome measures (ie, concerns about online use, desired components of online program, level of interactivity, and reasons to use an online program).

### Regression Models

Multimedia Appendix 1 shows the complete results of the regression models with the three blocks of predictors and four outcome measures. Three distinct blocks were used to model predictors of the four outcome measures. Each block was configured to include between four and five predictors to create balance. The first block contained the number of lessons the parent viewed using the DVD, comfort using computers, whether they preferred taking SFP online or attending a class, whether they wanted to track their skills practice on a computer or a handout, and whether they would practice the newly learned skills at home without a coach. Comfort using computers was significantly associated with the index summing concerns accessing SFP Online (*F*_2_=5.34, *P*=.006). Parents who reported being very comfortable using computers expressed fewer concerns (mean 1.85, SE 0.42) compared to parents reporting they were somewhat comfortable (mean 3.03, SE 0.64) or not very comfortable (mean 3.68, SE 0.90), albeit these differences were not significantly different from each other by the Tukey-Kramer post hoc multiple comparison test.

The second block included whether parents had access to a computer with internet service, whether they would use SFP Online even if they had a class, if they had a mobile or smartphone to view SFP Online, would they view SFP Online if they had a smartphone, and whether they would record their home practice assignments on a computer or smartphone. Parents with access to a mobile phone reported significantly fewer concerns (*F*_1_=10.94, *P*=.001; those owning a phone: mean 2.23, SE 0.63; those not owning one: mean 5.34, SE 1.07). Those wanting to view SFP on their mobile phone reported fewer concerns (*F*_1_=4.76, *P*=.03, mean 2.97, SE 0.72) for parents who could view SFP on a mobile phone compared with those who could not view SFP on a mobile phone (mean 4.59, SE 0.82). The third block included a measure asking parents how many lessons should be included in SFP Online, the proposed length of the individual lessons, whether fun games would help their family learn new skills, and whether family members should earn reward points when engaging with the online program. None of the individual predictors were significantly associated with technological concerns using SFP Online*.*

The next model examined the associations between the same three blocks and an index assessing important programmatic components to include in SFP Online. For the first block, ease of using computers was the only significant measure in the model (*F*_2_=4.07, *P*=.02). The post hoc multiple comparison showed that parents expressing some comfort using computers wanted significantly fewer lesson components (mean 16.67, SE 0.68) compared with parents feeling very comfortable (mean 18.53, SE 0.44). Parents wanting to view SFP Online using a mobile phone wanted more online components (*F*_1_=10.46, *P*=.002; mean 20.19, SE 0.75) compared with parents who did not want to access the online program using a mobile phone (mean 18.24, SE 0.86). No predictors in the third block were individually significant.

The third index captured desired interactive components to include in the new online program. In the first block, the number of SFP lessons completed on the DVD was significant (*F*_2_=5.87, *P*=.004). Parents who completed an intermediate number of lessons (4 to 6) wanted more interactive components (mean 12.31, SE 1.32) compared with parents who watched the most lessons (>6 lessons or more: mean 10.98, SE 0.98) and parents who watched the fewest lessons (0-3 lessons: mean 8.59, SE 1.04). The second block included one significant measure; whether parents desired to take a class even if they took SFP Online was significant (*F*_3_=4.58, *P*=.005). Parents desiring to use SFP Online even with a class wanted more interactive components (mean 12.07, SE 1.31) compared with parents stating “no” (mean 6.18, SE 2.74, *P*=.09), although these post hoc comparisons were not significant.

The final block included three significant predictors assessing the number of proposed lessons for the new online program (*F*_2_=4.14, *P*=.02), wanting SFP Online to include fun games (*F*_2_=5.01, *P*=.009), and wanting rewards built into the program (*F*_3_=2.82, *P*=.04). Parents viewing between 1 and 5 lessons wanted fewer interactive components (mean 10.18, SE 1.63) compared with parents viewing between 6 and 10 lessons (mean 12.95, SE 1.27). Parents stating “yes” to wanting fun games also reported wanting more interactivity (mean 12.82, SE 1.13) compared with those stating “maybe” (mean 10.53, SE 1.09).

The final model included the same three blocks individually predicting reasons (motivations) to use SFP Online. In the first block, only tracking SFP skills practice on the computer (versus handouts) was significant (*F*_2_=4.38, *P*=.02). Parents not wanting to track their skills practice reported significantly fewer reasons to use SFP Online (mean 6.48, SE 0.81) compared with those saying “yes” (mean 8.17, SE 0.53) or “maybe” (mean 8.30, SE 0.47). In the second block, only wanting to take SFP Online in conjunction with a class was a significant predictor (*F*_3_=5.11, *P*=.002). Parents stating they would probably not do the online class in addition to a group class reported significantly fewer reasons to use SFP Online (mean 4.56, SE 1.17) compared with those stating “maybe” (mean 8.49, SE 0.67) and “yes” (mean 8.77, SE 0.69). In the third block, wanting rewards while using the new online program was significant (*F*_3_=5.17, *P*=.003). Parents who said they probably did not want rewards through the online program reported significantly fewer reasons to use SFP Online (mean 5.05, SE 0.99) compared with parents who did want rewards (mean 8.05, SE 0.47).

We then repeated the block entry procedure using logistic regression with a binary measure asking parents whether they were interested in using a game-like version of SFP (response categories “maybe” and “yes, very interested” combined to 1; “not really” and “not interested at all” combined to 0 as the reference category). To avoid convergence problems (maximum likelihood estimates that were not trustworthy), we examined each predictor one by one and collapsed response categories for the measures, reducing the number of cells in the estimation process. We then combined all the significant predictor measures culled from the individual models into a single model, controlling for race and age. Based on the individual models, parents who preferred taking SFP online (attend class was designated the reference or comparison class) were almost nine times as likely to want a game-like experience than those who were undecided (likelihood ratio [LR] χ^2^_2_=8.8, *P*=.01; OR 8.88, 95% CI 1.06-74.48) to want a game-like experience. Those parents who were undecided were five times as likely (OR 5.10, 95% CI 1.02-25.29) to want a game-like experience compared with the reference class. Unadjusted, the C-statistic (a variant of Somers’ D) was 0.719, and the rescaled *R*^2^ (coefficient of determination) accounted for 16.6% of the variance.

Parents reporting they were very comfortable using computers were eight times as likely to be interested in a game-like online version of SFP (LR χ^2^_2_=8.8, *P*=.01; rescaled *R*^2^=16.6%, C-statistic=0.654; OR 7.90, 95% CI 1.18-52.97) compared with those reporting they were not at all comfortable using computers. Parents with mobile phones on which they could view SFP Online were 11 times as likely to want a game-like online version than parents lacking mobile phone access (LR χ^2^_1_=3.96, *P*=.046; OR 11.66, 95% CI 0.98-138.92; rescaled *R*^2^=8%, C-statistic=0.564). Parents who had a smartphone were six times as likely to want fun games than parents who did not have smartphones (LR χ^2^_1_=6.1, *P*=.01; OR 6.02, 95% CI 1.52-23.84; rescaled *R*^2^=12%, C-statistic=0.636). Parents who stated they would be likely or somewhat likely to record home practice assignments on their computer were 78% more likely to want a game-like version compared with parents who reported they would be somewhat unlikely and unlikely (LR χ^2^_1_=6.3, *P*=.01; OR 0.22, 95% CI 0.07-0.72; rescaled *R*^2^=12%, C-statistic=0.673).

A model with all the individually significant predictors combined fit well (LR χ^2^_6_=23.2, *P*<.001; rescaled *R*^2^=40%, C-statistic=0.846, Akaike information criterion=66.84, Hosmer-Lemeshow goodness-of-fit test, χ^2^_5_=1.0, *P*=.96). When all six predictors were modeled at once, only the measure of preferring the online version to a class was significant (LR χ^2^_2_=6.4, *P*=.04; OR 16.27, 95% CI 1.49-177.02); adjusting for race and age did not improve the model fit.

## Discussion

### Principal Findings

Thematic content analysis indicated three key areas of concern raised by all study participants when asked about designing an online version of the SFP: (1) desired content of the program, (2) logistics affecting program delivery, and (3) interactive features that can stimulate engagement. Interestingly, these themes reflected commonality in how consumers, agency stakeholders, and experts view the essential components of an online parenting skills training program that also involves youth drug prevention. Importantly, experts emphasized features consistent with cognitive behavioral principles, including skills practice, positive feedback, low-stakes failure exercises, and using reward structures (ie, proficiency “bars”) that help families move toward various programmatic benchmarks. They also outlined several implementation challenges, including program length and finding ways to use multimedia and interactive features to reinforce the focus and core active ingredients of the program.

Experts also felt the online program content should be realistic, simple, and use video examples to teach problem-focused coping skills, reflect the body of literature on risk and protective factors, and provide parents with ready solutions to their problems. The parents, in particular, stated that all this should be crafted using animation that can exemplify role-playing skills and reinforce lesson content. Experts also felt that SFP Online should stimulate “authentic” collaboration between parent and child and offer coaching, discussion boards, and virtual interactions—all with the goal of increasing engagement to maintain high exposure over time.

Agency staff agreed in principle with the experts and felt that the online version should maintain a skills-oriented approach and be configured around real family experiences. Given their familiarity delivering the group format of SFP, they emphasized preserving many of the current fun activities that work well in the group setting. From a logistical standpoint, they listed numerous features that would enhance SFP Online*,* including shorter sessions, graphics emphasized over text, dashboards for the entire family, text message reminders, home practice materials, printable worksheets, and a means for agency personnel to track family progress. They also mentioned that parents may still want to meet in person with a facilitator, even if they had online access, and that boosters might be essential to reinforce program content over time.

Parents felt very enthusiastic about helping to design the program content and delivery format for the online version. They wanted shorter sessions and state-of-the-art navigation features, with pop-up notifications, rewind capabilities, drop-down screens and scrollable information, graphic display with virtual animation, and realistic role plays. The parents who had the most prior exposure to SFP provided the most feedback on program components that they believed could work online. They felt the program should include bulletin boards, quizzes, pause-and-practice features, proficiency rewards, weekly blogs from experts, and virtual role plays with instruction. They also felt the online version should appeal to a culturally diverse population.

The consumer preference survey augmented the inductive procedures by adding more fine-grained information that helped to clarify what factors are associated with technological barriers, course content, interactivity, and motivations to use the online version. Interestingly, technology-savvy parents expressed fewer concerns about using an online program and showed a clear preference for interactivity and designing SFP Online like a serious educational game. The parents reluctant to track their performance online and those not wanting performance rewards were less inclined to want a Web-based platform to learn parenting skills. Mobile phone access was ubiquitous in this sample, underscoring the narrowing of the digital divide in terms of access to mobile technology and the potential for eventually creating a mobile app to complement SFP Online [54]. In all models tested, there was no evidence that any differences in the outcome could be attributed to race, gender, or age.

Even though the qualitative portion of the study reinforced tremendous overlap between the different groups, there were also unique contributions expressed by each group. For instance, agency stakeholders desired to find ways of connecting parent and child in the Web platform as this could be a strength of SFP Online. In addition, agency stakeholders were less inclined to address animation as crucial to the program, and more inclined to address interactive components that reinforce behavior change (ie, downloads, FAQs, discussion boards) and the use of technology to improve compliance (eg, text reminders). In this case, the focus of stakeholders appeared vested more in creating teachable moments and less focused on using technology to increase consumer involvement.

### Strengths and Limitations

Several limitations should be mentioned. The data are cross-sectional, and we do not know, for example, whether any of the computer and mobile phone use questions actually predict subsequent use of SFP Online. Moreover, the purposive sampling intentionally recruited parents with prior exposure to SFP, including having used the DVD or participating in the group format. This can introduce bias because many of the parents who attended classes but desired an online version wanted the option to attend a class based on their prior exposure. Having the opportunity to receive immediate in-person feedback on their performance may be an attraction that is hard to duplicate on the Web. In addition, the small sample size altered the model testing sequence to avoid overfitting models. As a result, we could not test incremental variance attributed to each block of predictors. Related to this, some of the statistical relations may not have achieved significance merely because the study was underpowered. This problem underscores the trade-off between obtaining qualitative data from a small but sufficient number of focus groups and wanting a large enough and appropriately powered sample to produce robust parameter estimates. Notwithstanding, there is a wealth of information gathered using a mixed methods approach, and we certainly benefited from employing these different strategies.

One strength of this study is that we solicited collaborative input from prospective consumers as part of an active partnership preceding the development of the online program. This made participants feel valued and that they could contribute to program development in a meaningful way.

### Conclusion

Curtailing the rising tide of addiction in America requires taking evidence-based family prevention programs to scale, which can best be achieved by providing high-quality, low-cost, online delivery. Creating an effective, online family-based prevention tool that parents and youth will enjoy and use requires knowledge of what parents and youth want to learn, how they want to acquire this knowledge, and what factors will keep them engaged so they can develop the appropriate skills and complete the course.

## Appendix

Multimedia Appendix 1 Results of consumer preference survey regression models.

## Abbreviations

NIDA: National Institute of Drug Abuse
NIH: National Institutes of Health
SFP: Strengthening Families Program
