# Supplementary material for: Formative Evaluation to Build an Online Parenting Skills and Youth Drug Prevention Program: Mixed Methods Study
Source: JMIR Form Res. 2019 Nov 5;3(4):e14906. doi: 10.2196/14906 (PMC6914279; doi:10.2196/14906)
Supplement: Multimedia Appendix 1 [file formative_v3i4e14906_app1.pdf]

**Table S1. Results of consumer preference survey regression models**												
Survey Items by Block	Concerns			Components			Interactivity			Reasons to Use		
**Block #1**[a]	beta[b]	SE	*P*	beta	SE	*P*	beta	SE	*P*	beta	SE	*P*
No. SFP DVD lessons watched												
0-3 lessons	.000[c]	---	---	.000	---	---	.000	---	---	.000	---	---
4-6 lesson	-.167	.64	.79	0.58	0.67	.39	3.72	1.22	**.01**	1.48	0.67	**.03**
>6 lessons	-.514	.42	.22	0.82	0.44	.06	2.39	0.80	**.01**	0.68	0.44	.12
Comfort using a computer												
Not very comfortable	.000	---	---	.000	---	---	.000	---	---	.000	---	---
Somewhat comfortable	-0.65	.97	.51	-2.22	1.03	**.03**	0.88	1.87	.64	-0.13	1.02	.89
Very comfortable	-1.83	.88	**.01**	-0.35	0.94	.71	2.29	1.70	.18	-0.29	0.92	.75
Preferred method of doing SFP												
Attend class	.000	---	---	.000	---	---	.000	---	---	.000	---	---
Online	-0.89	.49	.07	-0.92	0.52	.07	-1.23	0.94	.19	1.03	0.51	**.05**
Undecided	-0.33	.46	.47	-0.31	0.49	.52	-0.87	0.88	.33	0.49	0.48	.31
Prefer tracking skills practice on computer or smartphone												
Maybe	.000	---	---	.000	---	---	.000	---	---	.000	---	---
Probably Not	0.598	.68	.38	-0.08	0.72	.91	-0.41	1.30	.75	-1.82	0.71	**.02**
Yes	-0.45	.44	.31	0.92	0.47	.05	-0.64	0.85	.45	-0.13	0.46	.78
Practice at home no live family coach												
Maybe	.000	---	---	.000	---	---	.000	---	---	.000	---	---
No	0.81	.97	.40	-0.41	1.03	.69	-2.52	1.86	.18	-1.27	1.01	.21
Yes	0.33	.41	.41	0.72	0.43	.09	1.29	0.78	.10	0.70	0.42	.10
Model Fit (F, R^2, *P*)	$F_{(12,72)}$ = 1.82, R^2=23%, *P* = .06			$F_{(12,72)}$ = 2.01, R^2=25%, *P* = .04			$F_{(12,72)}$ = 2.40, R^2=28%, *P* = .01			$F_{(12, 72)}$ = 2.02, R^2=25%, *P* = .03		
**Block #2**	beta	SE	*P*	beta	SE	*P*	beta	SE	*P*	beta	SE	*P*
Access to computer w Internet												
No	.000	---		.000	---	---	.000	---	---	.000	---	---
Yes	-0.17	.82	.84	-1.32	0.86	.13	-0.64	1.63	.69	0.29	0.85	.73
Use *SFP Online* even with class												
Maybe	.000	---	---	.000	---	---	.000	---	---	.000	---	---
Probably Not	-0.32	1.11	.77	0.43	1.17	.71	2.95	2.22	.18	-3.93	1.16	**.01**
No	0.54	1.28	.67	-1.29	1.34	.34	-3.99	2.55	.12	-1.19	1.33	.37
Yes	0.41	0.44	.34	0.05	0.46	.90	1.89	0.87	**.03**	0.28	0.45	.54
Access to mobile phone to view SFP												
No	.000	---	---	.000	---	---	.000	---	---	.000	---	---
Yes	-1.63	0.72	**.02**	-1.73	1.17	.15	1.35	2.23	.54	-1.43	1.16	.22
Would view SFP with mobile phone												
No	.000	---	---	.000	---	---	.000	---	---	.000	---	---
Yes	1.63	0.72	**.03**	1.95	0.76	**.02**	1.00	1.44	.49	-0.42	0.76	.57

Survey Items by Block	Concerns			Components			Interactivity			Reasons to Use		
Would record home practices on computer using SFP Online												
Likely	.000	---	---	.000	---	---	.000	---	---	.000	---	---
Somewhat likely	-0.18	0.46	.70	-1.14	0.49	**.02**	-0.71	0.93	.45	0.07	0.48	.87
Somewhat unlikely	0.18	0.60	.77	0.16	0.63	.80	-1.34	1.20	.26	-1.19	0.63	.07
Unlikely	-0.60	0.73	.41	-1.08	0.76	.16	-1.36	1.45	.35	-1.22	0.76	.11
Model Fit (F, R^2, *P*)	$F_{(11, 73)}$ = 1.98, R^2=23%, *P* = .04			$F_{(11,73)}$ = 2.32, R^2=26%, *P* = .02			$F_{(11,73)}$ = 1.95, R^2=23%, *P* = .04			$F_{(11,73)}$ = 2.24, R^2=25%, *P* = .02		
**Block #3**	beta	SE	*P*	beta	SE	*P*	beta	SE	*P*	beta	SE	*P*
Suggested number of lessons for SFP Online												
11-15 lesson	.000	---	---	.000	---	---	.000	---	---	.000	---	---
6-10 lessons	0.14	0.45	.76	-0.37	0.45	.41	-0.18	0.81	.82	-0.07	0.43	.87
1-5 lessons	0.36	0.69	.60	-1.18	0.69	.09	-2.95	1.24	**.02**	-0.79	0.65	
Proposed session length												
13-20 minutes	.000	---	---	.000	---	---	.000	---	---	.000	---	---
9-12 minutes	0.78	0.52	.18	-0.95	0.57	.10	-0.93	1.02	.37	-0.57	0.54	.29
6-8 minutes	0.08	0.67	.89	0.10	0.67	.88	-0.36	1.19	.76	-0.93	0.63	.14
21-30 minutes	0.29	0.56	.61	-0.15	0.56	.78	0.45	0.99	.65	-0.94	0.53	.08
30 minutes or longer	1.22	0.77	.12	0.65	0.77	.40	0.26	1.37	.85	-1.23	0.72	.10
Program should include fun games												
Maybe	.000	---	---	.000	---	---	.000	---	---	.000	---	---
Probably Not	0.37	1.40	.79	0.72	1.40	.61	2.37	2.50	.34	0.51	1.32	.70
Yes	-0.75	0.48	.12	0.86	0.48	.08	2.28	0.85	**.01**	0.77	0.45	.10
Earn reward points after completing assignments												
Maybe	.000	---	---	.000	---	---	.000	---	---	.000	---	---
Probably Not	0.40	1.04	.70	-0.09	1.04	.93	-2.43	1.86	.19			
No	1.54	1.95	.43	1.91	1.95	.33	5.37	3.48	.13	2.37	1.84	.20
Yes	0.28	0.50	.57	0.82	0.50	.11	1.32	0.89	.14	1.01	0.47	**.04**
Model Fit (F, R^2, *P*)	$F_{(11,73)}$ = 0.84, R^2=11%, *P* =.60			$F_{(11,73)}$ = 1.98, R^2=23%, *P* =.04			$F_{(11,73)}$ = 2.72, R^2=29%, *P* = .00			$F_{(11,73)}$ = 2.81, R^2=29%, *P* = .00		

[a] All models controlled for race and age. To reduce model strain from a small sample size, we tested predictors for the linear regression models uniquely as independent blocks. [b] Regression weights are unstandardized. [c] Indicates reference or comparison level.
